# Community drivers of tuberculosis diagnostic delay in Kampala, Uganda: a retrospective cohort study

**DOI:** 10.1186/s12879-021-06352-9

**Published:** 2021-07-04

**Authors:** Rachel Mercaldo, Christopher Whalen, Robert Kakaire, Damalie Nakkonde, Andreas Handel, Juliet N. Sekandi

**Affiliations:** 1 Department of Epidemiology and Biostatistics, College of Public Health, University of Georgia, Athens, GA USA; 2 Center for the Ecology of Infectious Diseases, University of Georgia, Athens, GA USA; 3 Global Health Institute, University of Georgia, Athens, GA USA; 4 School of Public Health, Makerere University, Kampala, Uganda; 5 Health Informatics Institute, University of Athens, Kampala, GA USA

**Keywords:** Transmission, Care-seeking delay, Community contact delay

## Abstract

**Background:**

Recent approaches to TB control have focused on identifying and treating active cases to halt further transmission. Patients with TB symptoms often delay to seek care, get appropriate diagnosis, and initiate effective treatment. These delays are partly influenced by whom the patients contact within their community network. We aimed to evaluate the community drivers of diagnostic delay in an urban setting in Uganda.

**Methods:**

In this study we analyze data from a retrospective cohort of 194 TB patients in Kampala, Uganda. We characterized the patterns of contacts made by patients seeking care for TB symptoms. The main outcome of interest was total community contact delay, defined as the time patients spent seeking care before visiting a provider capable of diagnosing TB.

**Results:**

Visits to health providers without access to appropriate diagnostic services accounted for 56% of contacts made by cohort members, and were significantly associated with community contact delay, as were symptoms common to other prevalent illnesses, such as bone and joint pain.

**Conclusions:**

Education programs aimed at primary care providers, as well as other community members, may benefit case identification, by informing them of rarer symptoms of TB, potential for co-infections of TB and other prevalent diseases, and the availability of diagnostic services.

**Supplementary Information:**

The online version contains supplementary material available at 10.1186/s12879-021-06352-9.

## Background

Tuberculosis (TB) is one of the top ten leading causes of death worldwide, and the leading cause of death by a single infectious agent in 2019 [1]. While TB prevalence studies indicate that infected individuals may transmit tuberculosis bacilli before symptom onset [2], the majority of transmission occurs between the debut of symptoms and treatment initiation [3]. Contagiousness, as measured by bacillary numbers on sputum smears, increases with treatment delays [4]. In the absence of a broadly effective vaccine, control measures rely on shortening this transmission period through early diagnosis and treatment of active pulmonary disease [4, 5].

Globally, the ideal of prompt identification and treatment of TB is not yet a realized norm; in many settings there is a fraction of patients who only receive diagnosis and treatment after a prolonged delay [6]. An extensive body of literature is dedicated to studies of delays at both the patient and healthcare system levels, in a variety of incidence or socio-economic settings. These studies have reported numerous risk factors for delay, including comorbidities [7, 8], low access to healthcare [9–11], initial visits to low-level healthcare facilities with inadequate diagnostic abilities [12–14], age and sex [8, 15], and beliefs or misunderstandings about the disease [16–18]. These factors affect delay in one or more stages by increasing the duration of time spent 1) experiencing symptoms without seeking care, 2) searching for qualified practitioners, or 3) awaiting diagnosis following a visit to a qualified provider or facility.

Categories of diagnostic delay have been summarized as either *health system delay* or *patient delay* [19]. Here, *health system delay* refers to the time from first contacting a qualified TB provider to final diagnosis and treatment, and may be related to numerous factors including sex of the patient [3, 20] or symptom profiles [21–23]. *Patient delay* refers to both the individual’s delay in seeking care and the time spent contacting unqualified providers or social contacts. While many studies have combined these patient delay portions of the diagnostic pathway for analysis, they are two distinct periods in which behavior and other drivers of delay likely differ. In our present study, we divide patient delay into its two component periods. *Care-seeking delay* was defined as a participant’s symptomatic time prior to seeking care. We defined *community contact delay* as the time spent actively seeking care in the community. Within this latter period, an individual may seek advice or help from any member of their community. This could include social contacts such as family or workmates, hereby termed social contacts, non-TB providers such as primary-level health providers or herbal or religious healers.

**Fig. 1:**
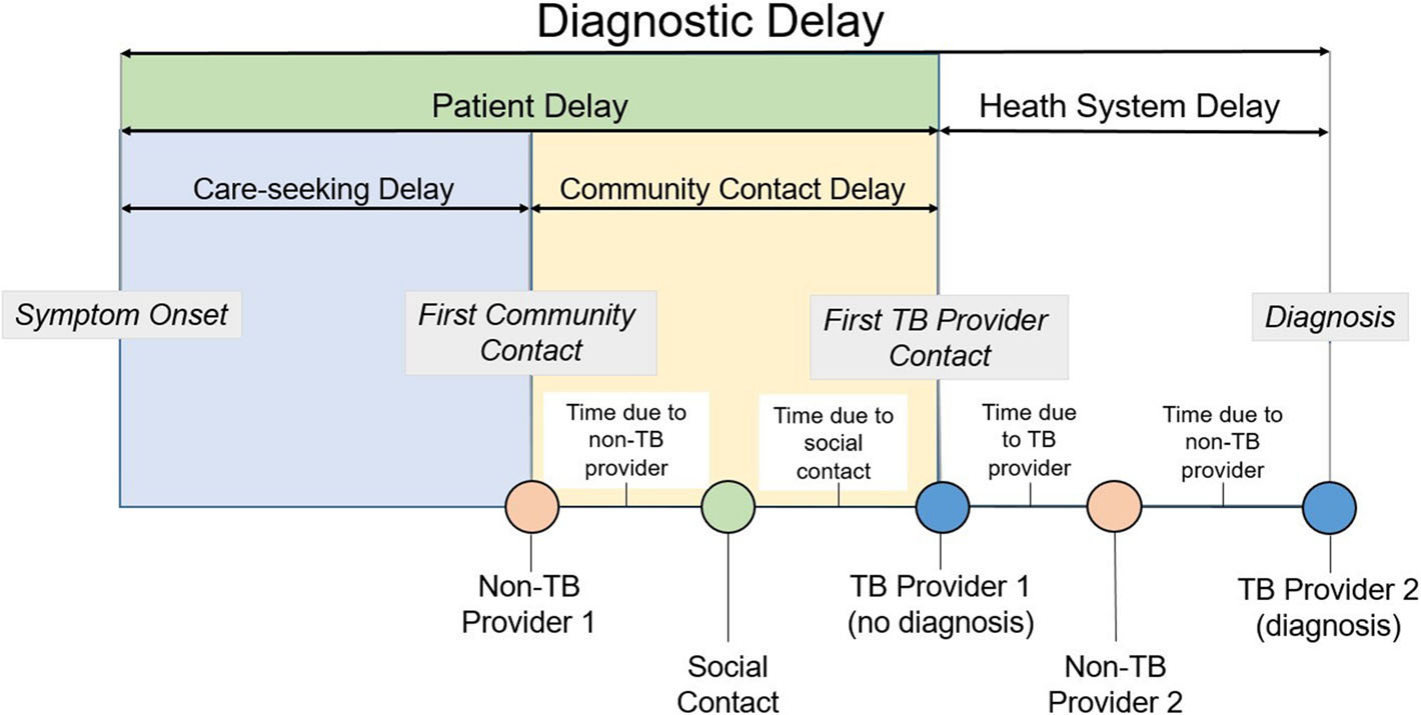
Conceptual Framework, with study definitions and an example of a realized diagnostic pathway. Diagnostic delay comprises patient and health system delays. Patient delay can be divided into two components: care-seeking and community contact delays. Within the community, a patient may contact several individuals, with each visit contributing time to delays (see text). In the example of a realized diagnostic pathway, the time contributed by social contacts is equal to the sum of the days between the social contact and the first TB provider, while the time contributed by non-TB providers was calculated as the number of days from non-TB provider 1 to the social contact. The time contributed by non-TB provider 2 is not counted toward contributions to community contact delay, as this visit took place during the health system delay period and so is counted toward health system delay

We maintained the definition of *healthcare delay* as the time from a participant’s first contact with a qualified TB provider to the point of final diagnosis. The different types of periods are summarized and illustrated in Fig. 1.

Previously, we conducted a retrospective cohort study (Diagnostic I) to quantify diagnostic delay in Kampala, Uganda, focusing on TB patients as members of a broader community of social contacts and health providers [5]. We examined patterns in delays to diagnosis among TB patients in urban health clinics, quantifying care-seeking delays and collecting detailed information regarding the social and provider contacts that participants make upon initiation of care-seeking. In a follow up study, Diagnostic II, we have expanded the questionnaire of the first study to examine additional factors contributing specifically to delay in the community. While other studies have focused on factors associated with decisions to seek care [24, 25] or on health system delays [3, 11, 21, 26], the present study examines factors associated with increased or decreased delay in the *community-contact* portion of the diagnostic pathway.

## Methods

We analyzed data from Diagnostic II, the second of two retrospective cohort studies conducted in Kampala, Uganda. This second study expanded on the methods of Diagnostic I, described previously [5].

### Ethical considerations

Written informed consent was obtained from all eligible participants. The study was approved by institutional review boards at the University of Georgia, Makerere University School of Public Health, and the Uganda National Council for Science and Technology. All methods were carried out in accordance with relevant human subjects guidelines and regulations.

### Study design, setting, and population

We conducted a retrospective cohort study among TB patients from January to November 2017. Participants were recruited at two public TB clinics located in Lubaga Division, and within 5–10 km of Kampala, Uganda’s capital city. The clinics are part of the government-funded public health system run by the Kampala Capital City Authority. Primary health care services, diagnosis and treatment of TB and other health conditions are provided free of charge. The estimated catchment population of the public clinics in Lubaga division is 400,000 persons. Additional health facility census information for the study area in 2017 is available from the United States Agency for International Development [27]. Eligible patients were consenting adults, eighteen years or older, who had been diagnosed with active pulmonary tuberculosis and who had initiated treatment within three months of the interview date. Participants were recruited at variable times after diagnosis and were interviewed to collect retrospective information on time of seeking care before diagnosis; this approach was previously deemed a suitable alternative to prospective cohort studies [28].

### Data collection and management

Data were collected in face-to-face interviews by trained interviewers using a structured questionnaire (available via our Github repository). The questionnaire was developed by a team of physicians, with expertise in TB, and epidemiologists. The original questionnaire used in our first study, Diagnostic I, was tested in a pilot study for accuracy, comprehension, and consistency of responses, with satisfactory results [5]. For Diagnostic II, the questionnaire was expanded to include items about participant knowledge about TB symptoms, experiences with and concerns about TB symptoms, prompts to seek care, and costs of reaching or obtaining health care. These variables were additions to the original items on HIV status, time of TB diagnosis, time of onset of symptoms, and duration of symptoms, as well as the detailed information about contacts made while seeking care. The complete list of variables is included as supplemental material.

Data were collected using standardized teleforms and scanned into a database using optical scanning software (TeleForms®). We preprocessed the raw data and engineered summary or comprehensive factors relevant to the analysis when applicable. All numeric variables were standardized—centered and scaled. All code and additional details are available as supplementary materials.

### Descriptive analysis

We calculated community contact delay as the time from first seeking care to first contacting a qualified TB provider. Qualified TB providers included government hospitals, government health centers, private hospitals, or other locations with TB diagnostic services.

For the analysis of these community delays, contacts were divided into two categories: social contacts and non-TB providers. Social contacts included spouses, parents, children, siblings, other relatives, coworkers, friends, and neighbors. Non-TB providers included herbal healers, drug stores, private clinics, or village health workers. The time contributed to a patient’s pathway was decomposed into steps between contacts, and each window of time was considered related to the most recent contact. In this way, the total community contact delay could be divided into the times specific to visits to contacts in each category. We calculated additional measures including the number and fraction of community network contacts in each category, as well as the total number of contacts and the total amount of time spent visiting contacts.

The outcome of interest was total community contact delay. As visits to non-TB providers were significant in the Diagnostic I study [5], a secondary analysis was included to explore factors associated with the number of community contact delay days spent in visits to non-TB providers.

### Statistical analysis

We fit linear regression models with each predictor individually, to investigate bivariate associations with community contact delay. Similarly, we fit bivariate regression models for each predictor for our secondary analysis, investigating the delay spent contacting non-TB providers.

Two final linear models were fit with Least Absolute Shrinkage and Selection Operator (LASSO) regularization and 10-fold cross validation—one each for the outcomes of (1) community contact delay and (2) the contribution of non-TB provider visits to community contact delay. The distribution of the residuals for full linear models with all predictors showed some skewness. Neither a log-transformation of the outcome nor use of Poisson distribution models improved the minor skew (see supplementary material), and linear regression was maintained for the final LASSO models. All analyses were conducted in R software (version 3.6.1) [29].

## Results

Table 1 reports characteristics of the study population. Of the 194 study participants, 177 (91.24%) were new TB patients, while only 17 (8.76%) were retreatment cases. The mean age of participants was 32 years (sd: 11.70 years), and 62.37% were male. There were 63 (32.47%) HIV positive participants and 129 (66.49%) who were HIV negative (Table 1).

**Table 1:** Baseline characteristics of participants in the Diagnostic II study

	(***n*** = 194)
**Sex**	
Female	72 (37.11%)
Male	121 (62.37%)
Missing	1 (0.52%)
**Age (Years)**	
Mean (SD)	32.00 (11.70)
Median [Min, Max]	28 [18, 82]
Missing	2 (1.03%)
**Marital Status**	
Currently married	69 (35.56%)
Not married	125 (64.43%)
**Monthly Income (UGX**^**a**^**)**	
Mean (SD)	294,000 (481,000)
Median [Min, Max]	200,000 [0, 5,000,000]
**TB Episode**	
New case	177 (91.24%)
Retreatment	17 (8.76%)
**HIV Status**	
Negative	129 (66.49%)
Positive	63 (32.47%)
Unknown	2 (1.03%)

^a^UGX, Ugandan Shillings. In the study year, 2017, the conversion rate for 1 US dollar was 3616.24 UGX

### Patterns of community contact delay

**Fig. 2:**
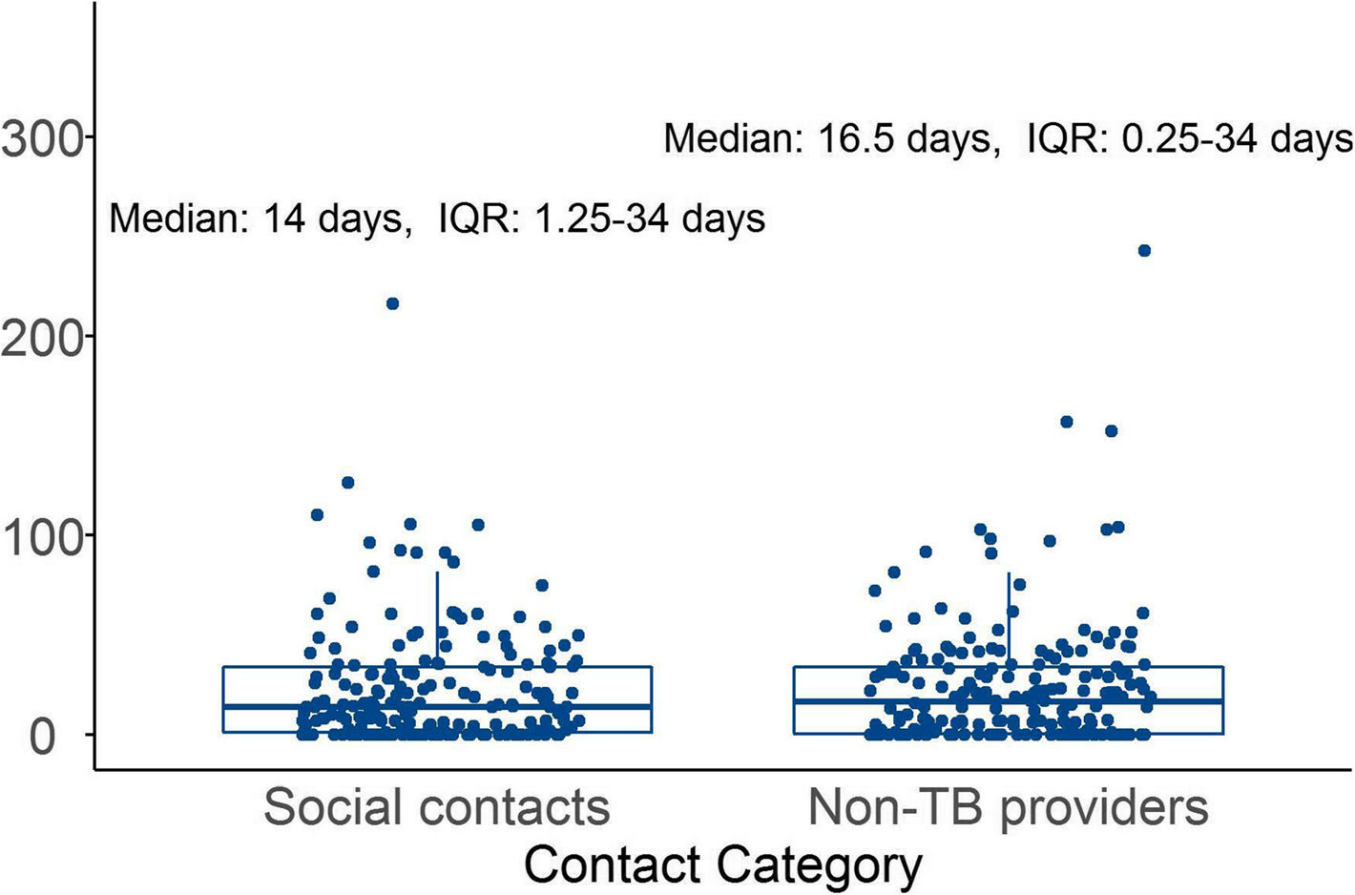
Time (in days) contributed to community contact delay by visits to social contacts or primary-level, non-TB providers in the Diagnostic II study. Each point represents one patient, with median and IQR shown

There were a total of 9014.69 days spent in the community contact period of the Diagnostic II cohort, with a median 33 days (IQR: 14–66.75) spent by each patient. The 194 participants reported visiting 895 contacts during this period. Of these, 397 (44.36%) were social contacts, while 498 (55.64%) were non-TB providers. Patients made a median 5 contacts within their community before reaching a qualified provider. Though the Diagnostic II cohort made approximately 25% more contacts with non-TB providers, the actual time contributed to overall community contact delay by visits to such providers (4625.56 days), was similar to that contributed by visits to social contacts (4378.13 days) (Fig. 2).

### Model results – community contact delay

Bivariate regression models of community contact delay were fit for all available predictors. As there were 49 predictors, the table of all results is too large to include in text and is available as supplemental material. Table 2 reports the results for the twelve significant variables (*p* < 0.05). A 10% increase in the proportion of contacts made to non-TB providers (rather than social contacts) was associated with an additional 1.20 days of delay in the community. Receiving cough medication was associated with 40.61 days of additional delay (*p* = 0.001), while each additional receipt of cough medication was associated with 11.35 days of delay (*p* = 0.0005). Suspicion that the illness was TB was associated with 16.36 fewer days of delay (*p* = 0.0143). Specific symptom knowledge or experiences were also associated with decreased delay: knowing that appetite loss or weight loss is a symptom of TB (18.45 fewer delay days, *p* = 0.0197), knowing that coughing blood or chest pain is a symptom of TB (14.23 fewer delay days, 0.0306), or seeking evaluation for TB due to night sweats or fever (15.15 fewer delay days, 0.0217)). Conversely, experiencing or feeling concern over bone or joint pain symptoms, or seeking care for bone or joint pain symptoms, was associated with increased delay (21.32 delay days and 39.27 delay days, respectively, *p* = 0.0032, *p* = 0.0118).

**Table 2:** Results of bivariate linear regression for significant (*p* < 0.05) predictors in models of community contact delay

Variable	Estimate	Pr(>|t|)	95% CI
Proportion of contacts in non-TB provider category (10% increments)^a^	1.20	0.0002	(0.57,1.82)
Number of times cough medication received	11.35	0.0005	(5.07, 17.63)
Total cost for care^b^	10.87	0.0009	(4.56, 17.18)
Suspected illness was TB	−16.36	0.0143	(−29.33, −3.40)
Received cough medication	40.61	0.0001	(20.10, 61.12)
Cough disrupted daytime activity	17.98	0.0070	(5.05, 30.91)
Knows appetite loss or weight loss is symptom of TB	−18.45	0.0197	(−33.82, −3.07)
Knows coughing blood or chest pain is symptom of TB	−14.23	0.0306	(−27.04, −1.43)
Experienced, or was concerned about, bone or joint pain	21.32	0.0032	(7.34, 35.29)
Sought care for bone or joint pain	39.27	0.0118	(8.98, 69.56)
Evaluated for TB due to bone or joint pain	25.67	0.0436	(0.90, 50.44)
Evaluated for TB due to night sweats or fever	−15.15	0.0217	(−27.98, −2.32)

^a^Coefficient should be read as the increase in delay days associated with each increase of 0.1 in the proportion of contacts that belong in the non-TB provider category

^b^UGX, Ugandan Shillings. In the study year, 2017, the conversion rate for 1 US dollar was 3616.24 UGX

In a final linear model with LASSO regularization and 10-fold cross-validation, fourteen variables were selected and are reported in Table 3. Experiencing or feeling concern about bone or joint pain was associated with 16.76 additional delay days. Seeking care for coughing blood or chest pain was also associated with an increase of 0.66 delay days, though the participant knowing these are symptoms of TB was associated with decreased delay (2.73 fewer days). Evaluation for TB due to night sweats or fever was associated with 7.81 fewer delay days, while finding no relief from self-medicating was associated with 3.37 fewer delay days. Receiving cough medication was associated with 21.49 additional days of delay. Notably, a 10% increase in the proportion of contacts in the non-TB provider category was associated with only 0.58 additional days of delay in the final model.

**Table 3:** Results of linear regression (with LASSO regularization) models of community contact delay

Model R^**2**^: 0.29	
Variable	Estimate
Experienced, or was concerned about, bone or joint pain	16.76
Sought care for coughing blood or chest pain	0.66
Evaluation for TB due to night sweats or fever	−7.81
No relief from self-medication prompted care-seeking	−3.37
TV/Radio advertisement prompted care-seeking	5.22
Knows coughing blood or chest pain is a symptom of TB	−2.73
Knows appetite loss or weight loss is a symptom of TB	−4.95
Received cough medication	21.49
Cough disrupted daytime activity	3.88
Suspected illness was TB	−8.96
Bought supplements	−0.52
Diagnosis location - outside Rubaga	−0.29
Proportion of contacts in non-TB provider category (10% increments)^a^	0.58
Total cost of reaching care^b^	4.45

^a^Coefficient should be read as the increase in delay days associated with each increase of 0.1 in the proportion of contacts that belong in the non-TB provider category

^b^UGX, Ugandan Shillings. In the study year, 2017, the conversion rate for 1 US dollar was 3616.24 UGX

### Model results – days of delay spent in visits to non-TB providers

As visits to contacts in the non-TB provider category was found significant in our original study, Diagnostic I [5], we also analyzed factors associated with the time spent in visits to these contacts in the present study. In bivariate analysis (Table 4), receipt of cough medication was associated with 23.33 days of delay following visits to these providers, (*p* = 0.0016). Each time medication was received was associated with 8.85 delay days (*p* = 0.0001). Increases in total cost of reaching care (in terms of Ugandan shillings, centered and scaled) was significantly associated with increased delay (6.56 days, *p* = 0.0036). Symptom knowledge was associated with decreased delay: 10.77 fewer days for knowing appetite loss or weight loss is a symptom of TB (*p* = 0.0481) and 9.74 fewer days for knowing coughing blood or chest pain was a symptom of TB (*p* = 0.0313). Seeking care for bone or joint pain was associated with 37.38 additional delay days (*p* = 0.0004), while being evaluated for TB due to bone or joint pain was associated with 21.25 additional days of delay following visits to non-TB providers (*p* = 0.0148).

**Table 4:** Results of bivariate linear regression for significant (*p* < 0.05) predictors in models of delay spent in visits to non-TB providers

Variable	Estimate	Pr(>|t|)	95% CI
Number of times cough medication received	8.85	0.0001	(4.54, 13.16)
Total cost for care^a^	6.56	0.0036	(2.19, 10.93)
Suspected illness was TB	−9.89	0.0350	(−19.02, − 0.76)
Received cough medication	23.33	0.0016	(9.06, 37.59)
Knows appetite loss or weight loss is symptom of TB	−10.77	0.0481	(−21.38, −0.16)
Knows coughing blood or chest pain is symptom of TB	−9.74	0.0313	(−18.54, −0.94)
Sought care for bone or joint pain	37.38	0.0004	(16.88, 57.87)
Evaluated for TB due to bone or joint pain	21.25	0.0148	(4.31, 38.19)

^a^UGX, Ugandan Shillings. In the study year, 2017, the conversion rate for 1 US dollar was 3616.24 UGX

In a final linear model with LASSO regularization and 10-fold cross-validation, sixteen variables were selected and are reported in Table 5. A number of these factors are related to symptoms: experiencing or being concerned about cough-related symptoms or malaise were associated with 3.63 and 3.96 fewer days of delay, respectively, following visits to non-TB providers. Seeking care for bone and joint pain was associated with 21.98 additional delay days, while seeking care for cough symptoms was associated with 0.73 additional days. Symptom knowledge and the participant’s suspicion that the illness was TB were once more associated with decreasing delays, and receipt of cough medication again associated with increasing delays (Table 5).

**Table 5:** Results of linear regression (with LASSO regularization) models of delay spent in visits to non-TB providers

Model R^**2**^: 0.27	
Variable	Estimate
Experienced, or was concerned about, coughing blood or chest pain	−3.63
Experienced, or was concerned about, malaise	−3.96
Sought care for bone or joint pain	21.98
Sought care for coughing blood or chest pain	0.73
Evaluation for TB due to night sweats or fever	−0.53
Knows coughing blood or chest pain is a symptom of TB	−3.32
Knows appetite loss or weight loss is a symptom of TB	− 0.78
Received cough medication	5.03
Suspected illness was TB	−4.10
Someone other than participant expressed concern about symptoms	−1.18
Bought supplements	−4.51
Age (years)	0.04
Marital status - currently married/cohabiting	−0.06
TB episode - first episode	−0.05
Total cost of reaching care^a^	7.85

^a^UGX, Ugandan Shillings. In the study year, 2017, the conversion rate for 1 US dollar was 3616.24 UGX

## Discussion

In this exploratory analysis of the Diagnostic II retrospective cohort data, we found that tuberculosis patients sought care from their community contacts for a median of 33 days before contacting a health care professional at a government-designated TB service clinic. The 194 participants spent 9015 cumulative days actively seeking care in the community.

Ideally, patients should seek care from specialized TB providers upon recognizing symptoms. In the Diagnostic I study, we suggested that patients, possibly unaware of the cause of their illness, may first seek care through another care provider or social contact who then refers them to the appropriate diagnostic location. In the present study, we found that patients made a median 5 such contacts before reaching a qualified provider.

Previous studies have shown that patients cycle through repeated visits to lower-level, primary, health providers [3, 27, 30], and our Diagnostic I study shows a significant portion of diagnostic delay, within the community contact portion of the pathway, was spent in visits to non-TB providers. We have recommended interventions targeting non-TB providers to reduce these delays [5]. In the present study, we show that, while the actual delay time due to non-TB providers was similar to that of social contacts, patients made 25% more visits to non-TB providers.

In a secondary analysis, we focused on the time spent in visits to non-TB providers within the Diagnostic II population. We found that a symptom attributable to other prevalent febrile diseases, bone and joint pain, was significantly associated with the time spent in visits to non-TB providers. Further, bone and joint pain symptoms, as well as cough-related symptoms, were selected in a linear model with LASSO regularization. As these symptoms are rare for TB, it is possible that patients or non-TB providers first attempt treatment for other prevalent febrile diseases, such as malaria, for which such symptoms are more common. With these results, similar to those found in other studies [3, 21], we maintain the recommendation that non-TB providers complete continuing education emphasizing TB screening, even in such cases when malaria, typhoid, or other febrile illnesses are suspected. Additionally, they should be encouraged to refer patients to proper diagnostic locations or recommend them for follow-up, possibly through active case finding.

We acknowledge that coinfection of TB and other illnesses that cause fever, such as HIV or malaria, is certainly a possibility. The present study is not able to identify such cases, or the cause of symptoms common to other diseases (such as bone and joint pain in malaria). Further research on the prevalence of coinfection and associated symptom profiles would improve education efforts.

We assumed that each contact in a patient’s diagnostic pathway was a separate, non-overlapping event, and so our calculated community contact delay may be overestimated. Additionally,

our data collection relied on patient-reported details, such as the length of time they experienced symptoms or the time between contacts, and as such is subject to recall bias. We recruited participants who had been diagnosed with TB within only three months of their interview, to decrease bias in this area.

While many studies have analyzed data on patient delays to diagnosis, our analysis focuses on a unique period in the diagnostic pathway—the time spent seeking care and contacting members of the community until a final diagnosis is reached. Recommendations for shortening delay at this stage may differ from those made to shorten care-seeking delays (when symptomatic patients have not yet begun to seek care). Community-based TB programs often focus on recognition of common TB symptoms—chronic cough, weight loss, night sweats, and fever—and encourage those with symptoms to visit health facilities or otherwise seek care. Our results suggest that further delays, once the patient is engaged in seeking care, may depend on interactions in the community and, particularly, with lower-level healthcare providers. Education efforts targeted for specific audiences (non-TB providers versus social contacts or the patients themselves) might focus on rarer symptoms of TB, or the wisdom of visiting TB diagnostic locations despite recognizing symptoms more common to other prevalent diseases. Some models of creating mass awareness about TB have been proposed and used elsewhere [31]. To facilitate appropriate actions that shorten community contact delays, improved point of care (POC) diagnostic TB tests that are delivered at the most decentralized levels of care where the patients make the initial contact with the non-TB provider health system, as well as within the community, are needed [32]. The use of POC at community level would minimize any barriers or further delays in case detection that are introduced during the referral process to TB service centers.

## Conclusion

The Diagnostic II cohort spent 9015 cumulative days actively seeking care in the community.. Patient recognition of TB symptoms was significantly associated with decreased delays, while seeking care from non-TB providers was associated with slightly increased delay. Continuing education for both the community and providers, and improving point of care (POC) diagnostics within local communities, may benefit symptom recognition and case identification, and decrease overall diagnostic delays.

## Supplementary Information

**Additional file 1.**

## Data Availability

All materials are available at https://github.com/RachelMercaldo/CommunityDriversDelay.
