# Supplementary material for: Community drivers of tuberculosis diagnostic delay in Kampala, Uganda: a retrospective cohort study
Source: BMC Infect Dis. 2021 Jul 4;21:641. doi: 10.1186/s12879-021-06352-9 (PMC8255016; doi:10.1186/s12879-021-06352-9)
Supplement: Supplementary file 1 — Additional file 1. [file 12879_2021_6352_MOESM1_ESM.pdf]

Supplemental material to accompany Community drivers of tuberculosis diagnostic delay in Kampala, Uganda: A retrospective cohort study.

1. Complete list of all predictors (highlighted are on continuous scale)

Supplemental Table 1: List of predictors included in analysis.

Age	Experienced, or was concerned about, appetite loss or weight loss - No/Yes
Monthly income*	Experienced, or was concerned about, bone or joint pain - No/Yes
Proportion of contacts in non-TB provider category (10% increments)**	Experienced, or was concerned about, coughing blood or chest pain - No/Yes
Number of symptoms prompting care-seeking	Experienced, or was concerned about, malaise - No/Yes
Number of cough symptoms prompting care-seeking	Experienced, or was concerned about, night sweats or fever - No/Yes
Number of times cough medication received	Bloody sputum prompted care-seeking - No/Yes
Total cost for care*	Frequent cough prompted care-seeking - No/Yes
Daily earnings lost while seeking care*	Reported no prompts to seek care
Sex	No relief from self-medication prompted care-seeking - No/Yes
Marital status	Painful cough prompted care-seeking - No/Yes
Employed - No/Yes	TV/Radio advertisement prompted care-seeking - No/Yes
Residence – division of residence	Sought care for appetite loss or weight loss - No/Yes
Smoking status	Sought care for bone or joint pain - No/Yes
HIV status	Sought care for coughing blood or chest pain - No/Yes
First TB episode – No/Yes	Sought care for malaise - No/Yes
TB was suspected - No/Yes	Sought care for night sweats or fever - No/Yes
Received cough medication - No/Yes	Evaluated for TB due to appetite loss or weight loss - No/Yes
Bought supplements - No/Yes	Evaluated for TB due to bone or joint pain - No/Yes
Cough disrupted daytime activity - No/Yes	Evaluated for TB due to coughing blood or chest pain – No/No/Yes
Cough disrupted Night - No/Yes	Evaluated for TB due to malaise - No/Yes
Knows appetite loss or weight loss is symptom of TB - No/Yes	Evaluated for TB due to night sweats or fever - No/Yes
Knows bone or joint pain is symptom of TB - No/Yes	Diagnosis location (private or government clinics or hospitals)
Knows coughing blood or chest pain is symptom of TB - No/Yes	
Knows malaise is symptom of TB - No/Yes	
Knows night sweats or fever is symptom of TB - No/Yes	

**UGX, Ugandan Shillings. In the study year, 2017, the conversion rate for 1 US dollar was 3616.24 UGX.*

**Coefficient should be read as the increase in delay days associated with each increase of 0.1 in the proportion of contacts that belong in the non-TB provider category

2. Feature engineering during the analysis step:

During data cleaning, and analysis, variables were cleaned and summarized to reduce the dataset to a meaningful 49 predictors (listed above). The majority of the changes were made to combine information for each participant's numerous contacts to calculate total community contact delay.

Below are details of how some variables underwent additional recoding during the analysis step of the study.

Code for changes made during the data analysis step, rather than the data cleaning step, is included in the data analysis R file, while the data cleaning code is in a separate file.

Changes:

All numeric variables were centered and scaled.

NAs in delay time categories were set to 0 days, as these indicate no time spent in that portion of the pathway.

Marital status levels of "Widowed", "Separated/Divorced", and "Single/Never Married" were set as "Not currently married", in contrast to "Married/Cohabiting"

For analysis, unknown HIV status was coded as NA (for 2 participants)

Smoking status was coded as "Current or former smoker" versus "Never smoked".

Diagnosis location was coded as "Government" versus "Private" facilities.

Divisions of residence included "Central", "Rubaga", "Kawempe","Makindye", "Nakawa" and "Other", but was coded as "Rubaga" (location of study clinics and most common response) versus "Other".

3. Results of all bivariate analyses (significant results highlighted)

Supplemental Table 2: Complete results of bivariate linear regression for all included predictors

Variable	Estimate	Std. Error	Pr(>|t|)
**Outcome: Community contact delay**			
Age	2.5286	3.3339	0.4491
Monthly Income*	6.1475	3.2833	0.0627
Proportion of contacts in non-TB provider category (10% increments)**	1.1974	3.1985	0.0002
Number of symptoms prompting care-seeking	-3.8546	3.3015	0.2444
Number of cough symptoms prompting care-seeking	-2.8465	3.3068	0.3904
Times Cough Medication Recevied	11.3507	3.2024	0.0005
Total Cost for Care*	10.8727	3.2189	0.0009
Daily Earnings Lost*	3.7072	3.3113	0.2643
Sex - Male	-2.3080	6.8661	0.7371
Marital Status - Currently married	-7.2428	6.8833	0.2940

Employed - Yes	13.4140	8.1932	0.1032
Residence - Rubaga	*Baseline*		
Residence - Other	6.5002	6.5584	0.3229
Smoking Status - Never Smoked	*Baseline*		
Smoking Status - Current or former smoker	13.4246	7.9427	0.0926
HIV Status - Positive	0.4517	7.1089	0.9494
First TB - No	-13.7334	11.6451	0.2397
TB was Suspected - Yes	-16.3629	6.6157	0.0143
Others Expressed Concern about Symptoms - Yes	1.5097	6.6840	0.8216
Received Cough Medication - Yes	40.6103	10.4649	0.0001
Bought Supplements - Yes	-10.1309	7.3693	0.1708
Cough Disrupted Daytime Activity - Yes	17.9784	6.5977	0.0070
Cough Disrupted Night - Yes	6.5899	7.8770	0.4039
Coughed More in Night or Day - Night	-5.0416	8.0862	0.5337
Knows Appetite loss or Weight loss is Symptom of TB - Yes	-18.4473	7.8439	0.0197
Knows Bone or joint pain is Symptom of TB - Yes	16.6018	26.7553	0.5357
Knows Coughing blood or chest pain is Symptom of TB - Yes	-14.2328	6.5321	0.0306
Knows Malaise is Symptom of TB - Yes	-12.7396	17.6957	0.4724
Knows Night sweats or fever is Symptom of TB - Yes	-6.7087	7.2766	0.3577
Experienced, or was concerned about, Appetite loss or Weight loss - Yes	10.5792	7.9873	0.1869
Experienced, or was concerned about, Bone or joint pain - Yes	21.3163	7.1286	0.0032
Experienced, or was concerned about, Coughing blood or chest pain - Yes	-13.0938	19.0649	0.4930
Experienced, or was concerned about, Malaise - Yes	10.4962	6.9875	0.1347
Experienced, or was concerned about, Night sweats or fever - Yes	-7.4541	11.6748	0.5239
Bloody Sputum Prompted Care-Seeking - Yes	-6.4914	11.1081	0.5597
Frequent Cough Prompted Care-Seeking - Yes	-2.6543	6.6666	0.6910
Reported No Prompts to Seek Care	-31.2900	32.6377	0.3389
No Relief from Self-Medication Prompted Care-Seeking - Yes	-7.8980	7.8707	0.3169
Painful Cough Prompted Care-Seeking - Yes	-2.4608	8.1664	0.7635
TV/Radio Advertisement Prompted Care-Seeking - Yes	8.3038	8.3050	0.3186
Sought Care for Appetite loss or Weight loss - Yes	-5.5524	10.2147	0.5874
Sought Care for Bone or joint pain - Yes	39.2711	15.4537	0.0118
Sought Care for Coughing blood or chest pain - Yes	10.0009	7.2569	0.1698

Sought Care for Malaise - Yes	4.9782	19.0849	0.7945
Sought Care for Night sweats or fever - Yes	-4.9865	7.0192	0.4783
Evaluated for TB due to Appetite loss or Weight loss - Yes	-1.4515	7.4599	0.8459
Evaluated for TB due to Bone or joint pain - Yes	25.6708	12.6358	0.0436
Evaluated for TB due to Coughing blood or chest pain - Yes	-4.3563	6.6017	0.5101
Evaluated for TB due to Malaise - Yes	-11.3390	19.0708	0.5528
Evaluated for TB due to Night sweats or fever - Yes	-15.1500	6.5472	0.0217
DX Location - Government facility	*Baseline*		
DX Location - Private facility	-7.2542	6.6927	0.2798

**Variable**	**Estimate**	**Std. Error**	**Pr(>|t|)**
**Outcome: Non-TB provider contribution to delay**			
Age (years)	3.3092	2.2830	0.1489
Monthly Income*	0.9407	2.2763	0.6799
Number of symptoms prompting care-seeking	-1.3697	2.2751	0.5479
Number of cough symptoms prompting care-seeking	-0.4274	2.2771	0.8513
Times Cough Medication Recevied	8.8505	2.1975	0.0001
Total Cost for Care*	6.5592	2.2275	0.0036
Daily Earnings Lost*	2.5244	2.2816	0.2699
Sex - Male	-3.3699	4.7143	0.4756
Marital Status - Currently married	-4.2241	4.7350	0.3735
Employment - Yes	5.9774	5.6612	0.2924
Residence - Rubaga	*Baseline*		
Residence - Other	-1.4318	4.6086	0.7564
Smoking Status - Never Smoked	*Baseline*		
Smoking Status - Current or former smoker	5.1891	5.4923	0.2460
HIV Status - Positive	-0.3654	4.8878	0.9405
First TB - No	-11.6065	7.9894	0.1479
TB was Suspected - Yes	-9.8881	4.6571	0.0350
Others Expressed Concern about Symptoms - Yes	-2.7862	4.6822	0.5525
Received Cough Medication - Yes	23.3260	7.2776	0.0016
Bought Supplements - Yes	-9.1911	5.0620	0.0710
Cough Disrupted Daytime Activity - Yes	8.8910	4.6009	0.0548
Cough Disrupted Night - Yes	4.3623	5.4149	0.4215

Coughed More in Night or Day - Night	-0.2144	5.5636	0.9693
Knows Appetite loss or Weight loss is Symptom of TB - Yes	-10.7675	5.4131	0.0481
Knows Bone or joint pain is Symptom of TB - Yes	3.8836	18.4065	0.8331
Knows Coughing blood or chest pain is Symptom of TB - Yes	-9.7387	4.4903	0.0313
Knows Malaise is Symptom of TB - Yes	-10.8044	12.1545	0.3752
Knows Night sweats or fever is Symptom of TB - Yes	-7.2979	4.9849	0.1448
Experienced, or was concerned about, Appetite loss or Weight loss - Yes	3.4654	5.5094	0.5301
Experienced, or was concerned about, Bone or joint pain - Yes	5.4139	4.9974	0.2800
Experienced, or was concerned about, Coughing blood or chest pain - Yes	-24.3259	13.0023	0.0629
Experienced, or was concerned about, Malaise - Yes	-2.0160	4.8288	0.6768
Experienced, or was concerned about, Night sweats or fever - Yes	-6.3856	8.0199	0.4269
Bloody Sputum Prompted Care-Seeking - Yes	-4.7599	7.6342	0.5337
Frequent Cough Prompted Care-Seeking - Yes	0.2810	4.5841	0.9512
Reported No Prompts to Seek Care	-9.4404	22.4767	0.6749
No Relief from Self-Medication Prompted Care-Seeking - Yes	-0.7855	5.4238	0.8850
Painful Cough Prompted Care-Seeking - Yes	0.6543	5.6143	0.9073
TV/Radio Advertisement Prompted Care-Seeking - Yes	-1.2755	5.7225	0.8239
Sought Care for Appetite loss or Weight loss - Yes	-4.0702	7.0203	0.5627
Sought Care for Bone or joint pain - Yes	37.3770	10.4569	0.0004
Sought Care for Coughing blood or chest pain - Yes	7.8583	4.9804	0.1162
Sought Care for Malaise - Yes	5.1280	13.1151	0.6962
Sought Care for Night sweats or fever - Yes	-1.1095	4.8303	0.8186
Evaluated for TB due to Appetite loss or Weight loss - Yes	-2.6396	5.1245	0.6071
Evaluated for TB due to Bone or joint pain - Yes	21.2483	8.6431	0.0148
Evaluated for TB due to Coughing blood or chest pain - Yes	-3.6927	4.5350	0.4165
Evaluated for TB due to Malaise - Yes	-9.2973	13.1031	0.4788
Evaluated for TB due to Night sweats or fever - Yes	-6.3304	4.5396	0.1648
DX Location - Government facility	*Baseline*		
DX Location - Private facility	0.6273	4.6140	0.8920

**UGX, Ugandan Shillings. In the study year, 2017, the conversion rate for 1 US dollar was 3616.24 UGX.*

**Coefficient should be read as the increase in delay days associated with each increase of 0.1 in the proportion of contacts that belong in the non-TB provider category

4. Residuals of models

There are minor skews associated with ~5 or 6 outlying values of delay in the linear model selected and presented in the text. Below, Q-Q plots for the selected linear model, as well as two other tested models (log-transforming the outcome and using a Poisson model) are shown. Q-Q plots are shown for the primary outcome models (community contact delay).

Supplemental figure 1: Q-Q Plot for selected linear model.

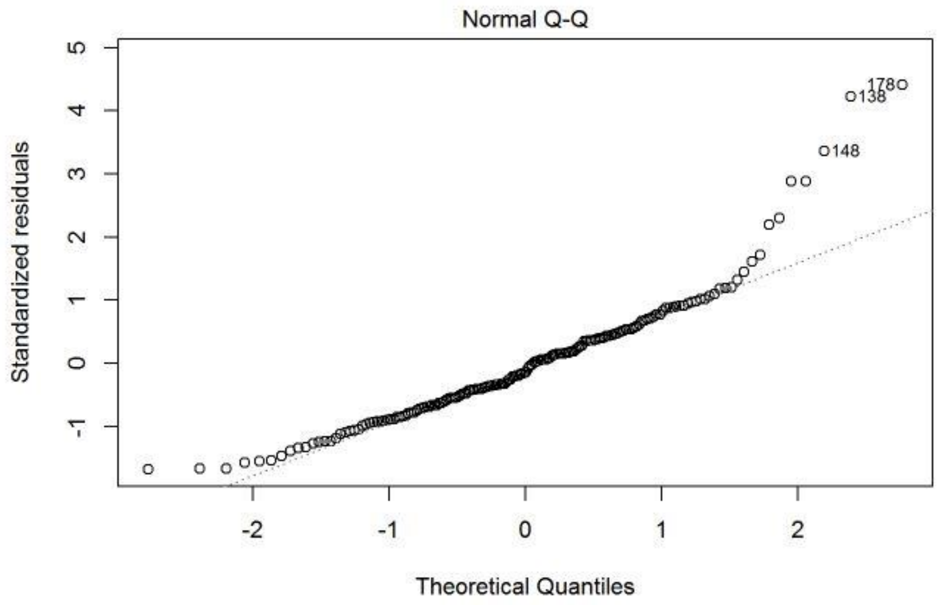

Supplemental figure 2: Q-Q plot for model with log-transformed outcome.

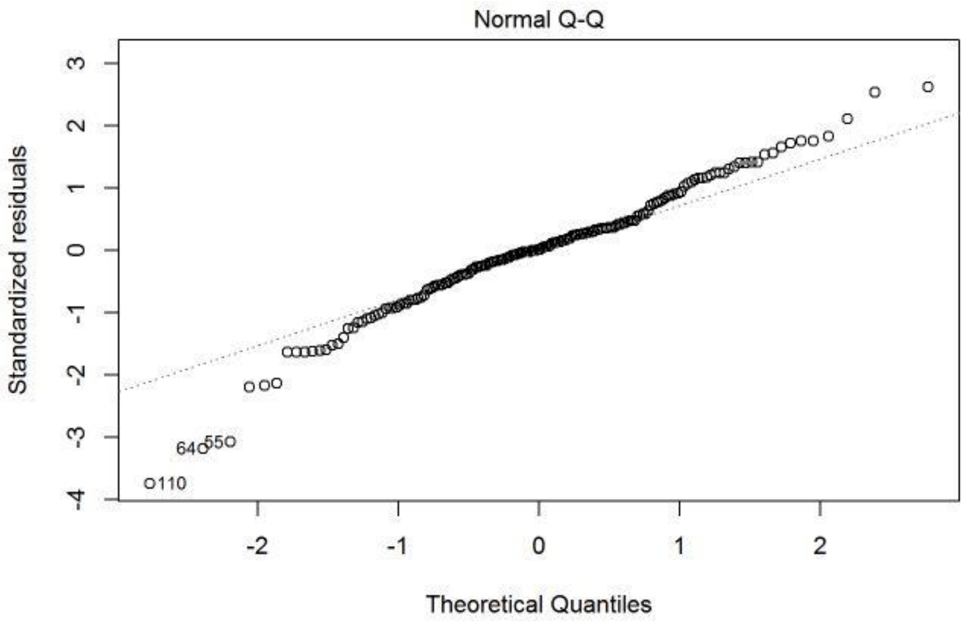

Supplemental figure 3: Q-Q plot for Poisson model.

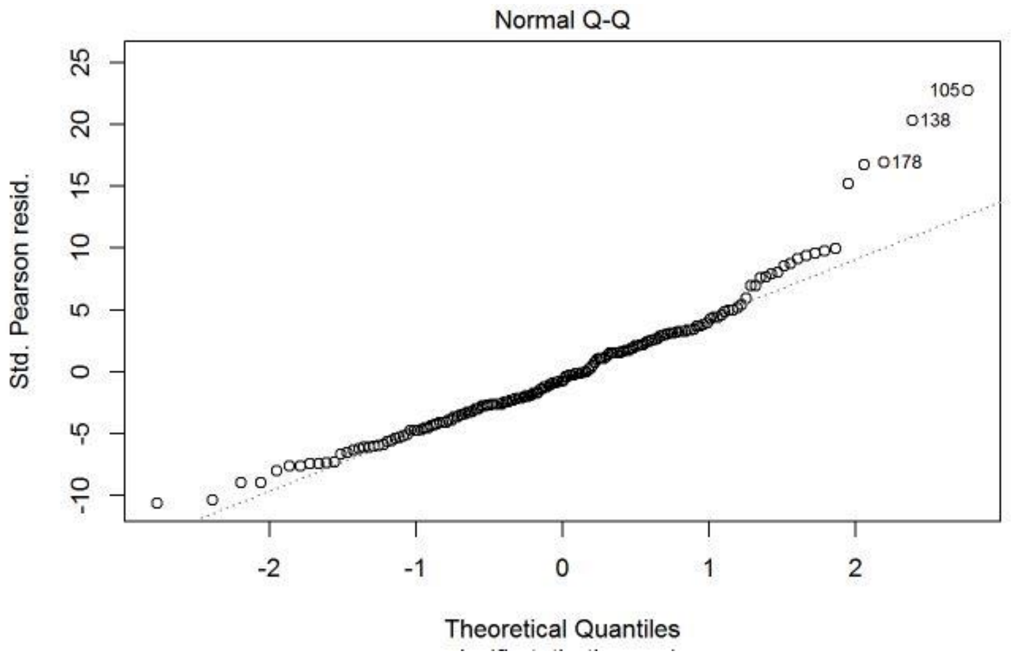

5. Results from previous study: Diagnostic I.

The results of our previous study, Diagnostic I, led to the expansion of the survey used to collect data and the second retrospective cohort study, Diagnostic II. Most of the results of Diagnostic I can be found in a previously published paper (1).

In addition to analyzing the Diagnostic II data to explore predictors of community contact delay, we also used the data of the original Diagnostic study to answer the same question. As different variables were collected in each study, we could not combine the data. We fit separate models for our primary outcome, community contact delay. The results of the Diagnostic II models are presented in the main text. Below, the results of the linear models of community contact delay with the limited set of predictors collected in Diagnostic I are shown.

Supplemental Table 3: Complete results of bivariate linear regression for all included predictors: Diagnostic I

Variable	Estimate	Pr(>|t|)
Age	-4.1556	0.2561
Income*	-0.2230	0.9513
Proportion of contacts in non-TB provider category (10% increments)**	3.2840	0.0000
Sex - Male	4.9130	0.5059
Marital Status - Currently married	16.0967	0.0327
Employed - Yes	-6.0350	0.5341
HIV Status - Positive	-0.2230	0.9510
First TB - No	-25.495	0.0027

Patient suspected illness was TB	-8.2600	0.3320
Family suspected TB - Yes	15.6091	0.0517
Knows how TB is transmitted - Yes	-0.0607	0.9930
Has chronic illness – Yes	21.2260	0.2510
Owns phone - No	4.5550	0.6220
Smoking status - current or former smoker	3.1450	0.7200

**UGX, Ugandan Shillings. In the study year, 2013, the conversion rate for 1 US dollar was approx. 2570 UGX.*
**Coefficient should be read as the increase in delay days associated with each increase of 0.1 in the proportion of contacts that belong in the non-TB provider category

Supplemental Table 4: Results of linear regression (with LASSO regularization) models of community contact delay: Diagnostic I

**Model R^2: 0.26**	
**Variable**	**Estimate**
Marital status – currently married	0.4746
Family suspected TB – Yes	0.0753
Income*	-3.7401
HIV Status – Positive	-2.9504
Owns phone – No	6.2131
Proportion of contacts in non-TB provider category (10% increments)**	2.9218

**UGX, Ugandan Shillings. In the study year, 2013, the conversion rate for 1 US dollar was approx. 2570 UGX.*
**Coefficient should be read as the increase in delay days associated with each increase of 0.1 in the proportion of contacts that belong in the non-TB provider category

6. References

1. Sekandi JN, Zalwango S, Martinez L, Handel A, Kakaire R, Nkwata AK, et al. Four Degrees of Separation: Social Contacts and Health Providers Influence the Steps to Final Diagnosis of Active Tuberculosis Patients in Urban Uganda. BMC Infect Dis. 2015;

Appendix 1: Diagnostic II Survey (English).

The English version of the survey used in the Diagnostic II survey:

MAKERERE UNIVERSITY-UNIVERSITY OF GEORGIA RESEARCH COLLABORATION
COMMUNITY HEALTH AND SOCIAL NETWORKS OF TUBERCULOSIS
DIAGNOSTIC PATHWAY II FORM_ENGLISH

Draft

Date of Interview ☐☐ / ☐☐ / 2 0 ☐☐
Day Month Year

1. In which division do you live? [Prompt]

☐☐ 01=Rubaga 04=Kawempe
02=Nakawa 05=Makindye
03=Central 88=Other ☐

2. How old are you? ☐☐ [Yrs]

3. Sex ☐ Male ☐ Female

3a. What is the highest level of education you attained?
- ☐ None
- ☐ Primary Level
- ☐ Secondary Level
- ☐ Post secondary specialization
- ☐ University degree

4. What is your marital status?

☐☐ 01 = Single/Never married
02 = Married/Cohabiting
03 = Separated/divorced
04 = Widowed

Specify

5. Are you employed ? ☐ Yes ☐ No

6. What is your main occupation? Whether you earn or don't earn an income from it

☐

7. On average, how much money do you earn in a month from all you jobs?
Shs ☐☐☐☐☐☐☐

8. What is the combined income of all household members including yours?
Shs ☐☐☐☐☐☐☐

9. Is this your first TB episode? ☐ Yes ☐ No

10. When did you receive your current TB diagnosis?
Please check the TB card
☐☐ / ☐☐ / 2 0 ☐☐
Day Month Year

11. Where was your current TB diagnosed?

☐☐ 01 = Private clinic
02= Private Hospital
03 = KCCA /Govt clinic
04= Gov't Hospital
88= Other,specify ☐

12. What method was used to diagnose your TB
- ☐ Sputum smear
- ☐ Chest X-ray
- ☐ GeneXpert
- ☐ Sputum culture

13. If smear was done, what was the result?
- ☐ Smear negative
- ☐ smear +++
- ☐ Smear +
- ☐ Smear scanty
- ☐ Smear ++
- ☐ N/A

14. When you first noticed your cough, did you contact anyone for advise? ☐ Yes ☐ No ☐ N/A

15. Who did you contact first for advise when you noticed that you had cough?
- ☐ Parent/sibling/relative
- ☐ Health provider at clinic
- ☐ Friend
- ☐ N/A
- ☐ Work mate
- ☐ Other,Specify ☐
- ☐ Freind (health worker)

16. After noticing cough or other symptoms what prompted you to take the first step to seek help?
- ☐ No relief from meds
- ☐ Uncertain
- ☐ Frequent coughing
- ☐ Other,Specify
- ☐ chest pain associated with cough
- ☐ N/A
- ☐ Coughed up blood

17. For how long did you have your cough, before you received this TB diagnosis? diagnosisomeone for advice or

Days ☐☐ Weeks ☐☐ Months ☐☐

18. For how long did you have these symptoms before you first sought advice from any person who is not a health provider about what to do?

Days ☐☐ Weeks ☐☐ Months ☐☐

Int Initials:	☐☐	Comp Date:	☐☐ / ☐☐ / 2 0 ☐☐
Rev Initials:	☐☐	Rev Date:	☐☐ / ☐☐ / 2 0 ☐☐
DMO Initials:	☐☐	DMO Date:	☐☐ / ☐☐ / 2 0 ☐☐
CD Initials:	☐☐	CD Date:	☐☐ / ☐☐ / 2 0 ☐☐
			Day Month Year

19. For how long did you have your cough or other symptoms before first sought treatment from a health provider or facility?

Days ☐☐ Weeks ☐☐ Months ☐☐

20. Did you receive any medicines for treatment of your cough? ☐ Yes ☐ No

21. If YES, how many times did you receive the medicines for your cough? ☐☐

22. List the sources of the medicines you received for your cough

- ☐ Drug store/pharmacy
- ☐ Traditional healer
- ☐ Mobile van/Vendor
- ☐ Private clinic
- ☐ Hospital
- ☐ Other (Specify) ☐

23. What prompted you to get health advise at the specific place where you received your TB diagnosis?

- ☐ No relief from medicine
- ☐ Persistent/worsening symptoms
- ☐ Other,Specify
- ☐ heard TB message on TV/radio
- ☐ Coughed up blood
- ☐ Referred by health provider at clinic
- ☐ refered by non health provider

Assessment of severity of cough

24. How would you rate the overall severity of your cough in the first two weeks after the onset?

- ☐ Mild cough
- ☐ Moderate cough
- ☐ Severe cough

25. How would you rate the overall severity of your cough before the TB diagnosis?

- ☐ Mild cough
- ☐ Moderate cough
- ☐ Severe cough

26. How would you rate the overall severity of your cough in the seven days preceeding your TB diagnosis?

- ☐ Mild cough
- ☐ Moderate cough
- ☐ Severe cough

28. Would you say your cough disrupted your daytime activities? ☐ Yes ☐ No

28. Would you say your cough disrupted your night time sleep? ☐ Yes ☐ No

29. Would you say your cough bothered you most at night or during day? ☐ Day ☐ Night

30. On the days when you coughed, how often did you cough?

- ☐ Rarely, Once a day
- ☐ Often (Many times but not every hour)
- ☐ Not so often (coughs occasionally)
- ☐ Almost all the time

31. How often did you cough in a week?

- ☐ Less than one day
- ☐ I dont recall
- ☐ 1-3 days/week
- ☐ 4-6 days/week
- ☐ Every day

32. Prior to the diagnosis of your TB, when you coughed did you cough up sputum? ☐ Yes ☐ No

33. Prior to the diagnosis of your TB, when you coughed did you see blood in your sputum?

☐ Yes ☐ No ☐ N/A

34. Prior to diagnosis of your TB, did your cough make you feel short of breath?

☐ Yes ☐ No

35. Prior to diagnosis of your TB, was your cough associated with chest pain?

☐ Yes ☐ No

Knowledge Assessment

36. Please, tell me some of the symptoms of TB that you knew about before receiving your diagnosis? [***Do not prompt respondents***] Mark all that apply

- ☐ Chronic cough
- ☐ Weight loss
- ☐ Loss of appetite
- ☐ Chest pain
- ☐ Malaise
- ☐ Bone/Joint pain
- ☐ Night sweats
- ☐ Fever
- ☐ coughing up blood
- ☐ None

37. Which symptoms below did you experience?

- ☐ Night Sweats
- ☐ Loss of Appetite
- ☐ Weight Loss
- ☐ Chest Pain
- ☐ Fever
- ☐ Malaise
- ☐ Bone/Joint pains
- ☐ Coughing blood
- ☐ Cough for more than 2 weeks
- ☐ N/A

Draft

38. Which symptoms above apart from cough concerned you? Check all that apply.

- ☐ Night Sweats
- ☐ Loss of Appetite
- ☐ Weight Loss
- ☐ Chest Pain
- ☐ Fever
- ☐ Malaise
- ☐ Bone/Joint pains
- ☐ Coughing blood
- ☐ N/A

39. Did you seek any healthcare for any of the symptoms above? ☐ Yes ☐ No ☐ N/A

40. If YES whch symptoms? Code 01 if present, 02 absent

- ☐ Night Sweats
- ☐ Loss of Appetite
- ☐ Weight Loss
- ☐ Chest Pain
- ☐ Fever
- ☐ Malaise
- ☐ Bone/Joint pains
- ☐ Coughing blood
- ☐ N/A

41. Which symptoms prompted you to seek evaluation for TB? Code 01 for Yes and 02 for No

- ☐ Night Sweats
- ☐ Loss of Appetite
- ☐ Weight Loss
- ☐ Chest Pain
- ☐ Fever
- ☐ Malaise
- ☐ Bone/Joint pains
- ☐ Coughing up blood
- ☐ N/A

42. Did you think you had TB before you were diagnosed?

☐ Yes ☐ No

43. Did someone else, such as a family member or friend, express concern about your illness before your TB diagnosis was made? ☐ Yes ☐ No

HIV Status and Cormobidities

44. What is your HIV status?

☐ Positive ☐ Negative ☐ Dont Know

45. Are you currently taking antiretroviral therapy?

☐ Yes ☐ No ☐ N/A

46. Have you ever been diagnosed with any of the following medical conditions(s)?

01 = Yes
02 = No
77 = Uncertain
88= Other

☐☐ Diabetes:

☐☐ Cancer

☐☐ Kidney disease:

☐☐ Liver disease:

☐☐ Asthma:

☐☐ Others(specify):

47. What is your current smoking status?

- ☐ Never smoked
- ☐ Current smoker
- ☐ Former smoker

48. For current smokers and past smokers, ask How long have you /did you smoke?

Days ☐☐ Weeks ☐☐ Months ☐☐

49. Do you drink alcohol? ☐ Yes ☐ No

50. If YES, for how long have you been drinking alcohol?

Days ☐☐ Weeks ☐☐ Months ☐☐

DIAGNOSTIC PATHWAY FORM_ENGLISH

Part B

Date of Interview [ ][ ] / [ ][ ] / [2][0][ ][ ]
Day Month Year

STUDY ID# [ ][ ][ ]

51. **Preamble to be read by Interviewer:** Now, think back about when you first noticed your symptoms of TB. We are interested in knowing the people you talked to about your symptoms and the places you may have sought care after you started feeling ill. Please tell me about what you did, who you confided in about your illness, or where you went to seek help before you had the final diagnosis of TB. **Remember**, this could be a family member, relative, friend, co-worker or any health provider or facility. I also want to know if you talked to the same person or visited the same place for help more than once.[Interviewer: Be sure to document separately each time a person or place is mentioned by the patient even if they are the same]

Now to begin, please tell me the first person/place you approached when your symptoms began. Remember, this may be a family member or friend. [Collect and Record]..........Good, can you tell me the NEXT person or place after that? [Collect & Record] [**Continue** with this line of questioning, with appropriate prompting and explanation, until patient indicates the place of diagnosis].

Contacts	Contact Person or Place	Contact Code	Lab code	Household member	Time btn contact	Unit for Time
Contact 1					0 0	0 0
Contact 2						
Contact 3						
Contact 4						
Contact 5						
Contact 6						
Contact 7						
Contact 8						
Contact 9						
Contact10						
Contact11						
Contact 12						
Contact 13						
Contact 14						
Contact15						
Contact 16						
Contact 17						

USE THE CODES BELOW TO COMPLETE TABLE ABOVE

Code for contact person or places visited ***QN: What person or place did you contact or visit for help/ treatment?***

01 = Herbal healer 02 = Drug store 03 = Gov't hospital 04 = Private hospital 05= Private clinic 06 = Gov't Heath center 07= Village health worker 08 = Spouse 09 = Parent 10= Brother/sister 11=Other relative 12=Co-worker 13 = Friend 14=Child 15= Neighbor 88=Other 99=Not applicable

Code for Household Member ***QN: Does the person listed live in the same household as the patient?***

01=Yes 02= No 99= Not applicable

Units for Time: ***QN: What time elapsed between the contacts......... [Refer to person or place listed above]***

01 = Days 02 = Weeks 03 = Months 77=Uncertain

Code for Lab existance ***QN: Does this place have any Laboratory***

01=Yes 02= No 99= Not applicable 77=Uncertain

Draft

52. When you contacted or visited {Name of place/ person} How much money did you spend on Travel, Phone calls, Laboratory tetsts, medications Food, Care giver.

	Facility/Person	Travel	Phone Calls	Lab Test/ CXR	Medication	Food	Caregiver
Cont. 1							
Cont. 2							
Cont. 3							
Cont. 4							
Cont. 5							
Cont. 6							
Cont. 7							
Cont. 8							
Cont. 9							
Cont. 10							
Cont. 11							
Cont. 12							
Cont. 13							
Cont. 14							
Cont. 15							
Cont. 16							
Cont. 17							

Explain briefly any unusual events in seeking care, costs or travels/movements as reported by the patient. For example if cost was incurred by someone else who is not the patient. This will help to provide additional information for data analysis and interpretation.

53. Before diagnosis, did you buy any supplements to your diet because of the TB illness, for example vitamins, meat, energy drinks, soft drinks or fruits?

☐ Yes ☐ No

54. If "YES", what kind of item was it?

Fruit ☐☐
Drinks ☐☐
Vitamins/herbs ☐☐
Meat ☐☐
Other Specify ☐☐ ______

55. How much in total did you spend on these items (s)

Shs ☐☐☐☐☐☐☐

56. On average, how much did you spend on your health when seeking care before your TB diagnosis?

Shs ☐☐☐☐☐☐☐

57. Before you were diagnosed with TB, did you ever miss days of school/work/housework because of TB

☐ Yes ☐ No

58. How many days did you miss work/school/housework due to your TB prior to your diagnosis

Days ☐☐☐

59. Did anyone stay home specifically to take care of you because you were sick?

☐ Yes ☐ No

60. How many fewer hours per day did you work?

☐☐

61. Before you were diagnosed, were you hospitalised for TB illness?

☐ Yes ☐ No

62. How long did you stay in hospital?

Days ☐☐☐

63. How much in total did you pay for your hospital stay

Shs ☐☐☐☐☐☐☐

Costs associated with TB illness

64. On average how much did you spend per month on your own healthcare BEFORE the onset of TB symptoms

Shs ☐☐☐☐☐☐☐

65. On average how much did you spend per month on your own healthcare after the onset of cough and other

Shs ☐☐☐☐☐☐☐

66. On average how much do you spend on your own healthcare NOW after your TB diagnosis?.

Shs ☐☐☐☐☐☐☐

67. We know that TB treatment is free, so since you got your TB diagnosis what did you spend you money for?

☐ Other medicines e.g Pyridoxine
☐ Transport to the clinic for medication
☐ Buying a drink/eating while at the clinic
☐ Supplementing nutritious foods at home
☐ other [Specify]
☐ N/A

68. Did you quit job or change jobs or quit school because of your TB illness?

☐ No
☐ YES- Quit school
☐ YES-Quit job
☐ YES-Changed job
☐ YES-Changed school
☐ N/A
☐ other [Specify] ______

69. Did you lose any daily income/earnings during this episode of illness?

☐ Yes ☐ No ☐ N/A

70.How much money would you say you lost per day before diagnosis?

Shs ☐☐☐☐☐☐☐

Ask patient to explain how income was lost?

Dissavings associated with TB illness Prior to Diagnosis

71. Did you borrow any money to cover costs due to illness? *[This includes costs for travel, phonecallls, medicines food, etc]* ☐ Yes ☐ No

72. How much money did you borrow?

Shs ☐☐☐☐☐☐☐

73. From whom did you borrow?

- ☐ Family member
- ☐ neighbors/friends/workmates
- ☐ Bank
- ☐ Private money lender
- ☐ other [specify] ________
- ☐ N/A

74. How much money total are you expected to pay or have you paid back for the amount you borrowed?

Shs ☐☐☐☐☐☐☐

75.When are you supposed to pay back this borrowed money? Code 00 in all boxes if not expected to pay?

Days ☐☐ Weeks ☐☐ Months ☐☐

76. Did your spouse/relative in your household borrow money to finance costs due to your current TB illness?

☐ Yes ☐ No

77. How much did your spouse/relative in your household borrow to pay for costs due to your current TB illness? Code 000000 in didnot borrow

Shs ☐☐☐☐☐☐☐

78. Have you sold any of your property to finance the costs of TB illness ☐ Yes ☐ No

79. If YES what did you sell?

- ☐ Land
- ☐ Livestock/Chickens
- ☐ Farm produce
- ☐ Household item
- ☐ Other [Specify] ________
- ☐ N/A

80. What is the estimated market value of the property you sold?Code 0000000 if no property sold

Shs ☐☐☐☐☐☐☐

81. List all items sold and the amount earned in Ug shs

ITEM	COST (UgShs)
	☐☐☐☐☐☐☐
	☐☐☐☐☐☐☐
	☐☐☐☐☐☐☐
	☐☐☐☐☐☐☐
	☐☐☐☐☐☐☐

82. Did any of your children below the age of 18 years work to finance costs due to your current TB illness?

☐ Yes ☐ No

83. Did any of your children below the age of 18 years miss school to take care of you or accompany you to seek care during your current TB illness?

☐ Yes ☐ No

84. Olina essimu eyomungalo ? ☐ Yes ☐ No (END HERE)

85. Oba 'YE" Kikka kya ssimu ki kyolina?

- ☐ Smart/Touch phone
- ☐ Non touch phone
- ☐ Both
- ☐ N/A

86. Omaze banga ki ne ssimu eyo?

Days ☐☐ Weeks ☐☐ Months ☐☐ Yrs ☐☐

87. Mbulira enamba zessimu sokozesezza okumala emyezi esatu egiyise nga wagiwandisa mumanya go.

Line one ☐☐☐☐☐☐☐☐☐☐

Line two ☐☐☐☐☐☐☐☐☐☐

88. Did patient consent for Cellphone research?

☐ Yes

☐ No
